# The Changing Landscape of Naive T Cell Receptor Repertoire With Human Aging

**DOI:** 10.3389/fimmu.2018.01618

**Published:** 2018-07-24

**Authors:** Evgeny S. Egorov, Sofya A. Kasatskaya, Vasiliy N. Zubov, Mark Izraelson, Tatiana O. Nakonechnaya, Dmitriy B. Staroverov, Andrea Angius, Francesco Cucca, Ilgar Z. Mamedov, Elisa Rosati, Andre Franke, Mikhail Shugay, Mikhail V. Pogorelyy, Dmitriy M. Chudakov, Olga V. Britanova

**Affiliations:** ^1^Shemyakin and Ovchinnikov Institute of Bioorganic Chemistry, Moscow, Russia; ^2^Center of Life Sciences, Skolkovo Institute of Science and Technology, Moscow, Russia; ^3^Istituto di Ricerca Genetica e Biomedica, Consiglio Nazionale delle Ricerche, Monserrato, Italy; ^4^Institute of Clinical Molecular Biology, Kiel University, Kiel, Germany

**Keywords:** aging, T cell receptor, naive T cells, immunosequencing, Rep-Seq, CDR3 repertoire

## Abstract

Human aging is associated with a profound loss of thymus productivity, yet naïve T lymphocytes still maintain their numbers by division in the periphery for many years. The extent of such proliferation may depend on the cytokine environment, including IL-7 and T-cell receptor (TCR) “tonic” signaling mediated by self pMHCs recognition. Additionally, intrinsic properties of distinct subpopulations of naïve T cells could influence the overall dynamics of aging-related changes within the naïve T cell compartment. Here, we investigated the differences in the architecture of TCR beta repertoires for naïve CD4, naïve CD8, naïve CD4^+^CD25^−^CD31^+^ (enriched with recent thymic emigrants, RTE), and mature naïve CD4^+^CD25^−^CD31^−^ peripheral blood subsets between young and middle-age/old healthy individuals. In addition to observing the accumulation of clonal expansions (as was shown previously), we reveal several notable changes in the characteristics of T cell repertoire. We observed significant decrease of CDR3 length, NDN insert, and number of non-template added N nucleotides within TCR beta CDR3 with aging, together with a prominent change of physicochemical properties of the central part of CDR3 loop. These changes were similar across CD4, CD8, RTE-enriched, and mature CD4 subsets of naïve T cells, with minimal or no difference observed between the latter two subsets for individuals of the same age group. We also observed an increase in “publicity” (fraction of shared clonotypes) of CD4, but not CD8 naïve T cell repertoires. We propose several explanations for these phenomena built upon previous studies of naïve T-cell homeostasis, and call for further studies of the mechanisms causing the observed changes and of consequences of these changes in respect of the possible holes formed in the landscape of naïve T cell TCR repertoire.

## Introduction

A diverse set of naïve T cell functions (1) and their antigenic receptors—T-cell receptors (TCRs) (2, 3)—protects us from a multitude of infectious and cancer hazards encountered throughout our lifespan. Furthermore, it essentially provides selection of the appropriate amplitude, type, localization, and duration of immune response. Human aging is associated with profound changes in T cell immunity (2–5), compromising our ability to withstand novel pathogens and manage chronic infections. It also dampens the effect of vaccination (6–8) and can lead to higher cancer susceptibility (9–12). These changes may further result in an imbalanced immune response that can develop into non-specific inflammation, provoking neurodegenerative and cardiovascular disorders, and to the loss of tolerance, leading to autoimmunity (3, 13–15). For the latter, a reduction of regulatory T cell (Treg) diversity (16, 17) could be a one of the causative factors.

With aging, accumulating clonal expansions of memory T cells caused by previously encountered antigens gradually begin to dominate in the available T-cell pool. This leads to a homeostasis characterized by a decreased number of naïve T cells, essentially shrinking the precious reservoir of diverse functions and antigenic specificities (2, 18–23). At the same time, thymus function progressively declines after puberty (24, 25), and drops sharply to a very low level after 40 years of age (4, 26). Along with diminished production of T cell progenitors by the bone marrow (27), this leads to a drop in generation of the so-called recent thymic emigrants (RTE)—the not fully mature (28, 29) form of naïve T cells, and thus in the replenishment of the mature naïve T cell pool (5, 26, 30).

The existing naïve T cells may still support their abundance and diversity for a prolonged period. In humans, both mature naïve T cells and—to a lesser extent—RTE-enriched CD45RA^+^CD31^+^ subset of CD4 T cells (30)—keep ability to proliferate on the periphery (31, 32). However, the number of allowed divisions is not unlimited. Prominent shortening of telomeres is observed in both CD31+ and CD31^−^ subsets (30) which eventually leads to a gradual, later avalanche, exhaustion of proliferation capacity and depletion of the naïve T cell pool (20, 33). Additionally, prolonged peripheral proliferation could also be associated with the functional deficiency of naïve T cells that fail to differentiate toward memory phenotype upon a specific antigenic challenge (3), although a recent cytokine profile study suggests that naïve T cells derived from elderly individuals retain their functionality and naiveté (26).

How uniform is the naïve T cell proliferation on the periphery remains questionable. Qi et al. demonstrated that both CD4 and CD8 naïve T cells gated as CCR7^+^CD45RA^high^CD28^+^ gain clonal expansions by the age of 70–85 years (34). This observation suggests that some of the naïve T cell clones are dividing more prominently than others. Furthermore, the most rapidly dividing ones could exhaust and extinguish more rapidly, while those dividing with a moderate rate could form the observed clonal expansions.

Importantly, the peripheral T cell proliferation may be dependent on the so-called “tonic signaling”—recognition of MHC complexes loaded with self antigens while surveying the peripheral lymphoid organs. Such contacts are transient and do not lead to classic T cell activation, but generate sub-threshold signals required for naïve T cell survival and proliferation (35–38).

The desirable (i.e., required to efficiently recognize foreign antigens within MHC) and allowed (i.e., not leading to self-recognition and autoimmunity) TCR affinity to self peptide–MHC complexes is set in the course of positive and negative thymic selection, respectively. The threshold range of such selection is not that narrow, thus naïve T cells that leave the thymus—initially as RTE—have a relatively wide range of self-reactivity. The produced pool of naïve T cells is, therefore, subjected to varying degrees of tonic TCR signaling (38). Therefore, peripheral proliferation of naïve T cells could be potentially biased toward preferential exhaustion of naïve T cell clones carrying TCRs with the highest affinity to MHC. Furthermore, naïve T cells bearing high affinity TCRs could also serve as a preferential source of antigen-responding clones (37) thus being the first one to transit from the naïve T cell pool.

Another factor that could contribute to the dynamics of naïve TCR repertoire landscape is the fate of the specific population of T cells produced in fetal period. We have earlier demonstrated that this subset may survive for decades and contribute to adult TCR repertoire (39). Their TCRs are characterized by a low number of nucleotides that are randomly added by TdT enzyme in the course of VDJ recombination (40, 41). Furthermore, these cells originate from a distinct population of hematopoietic stem cells and are characterized with generally higher proliferation potential (42). However, their fate among other naïve T cells in the elder age remains unexplored.

Altogether, there are number of factors that could shape the landscape of naïve T cell TCR repertoire with aging. To shed light on the nature of ongoing changes, we have focused on the comparative analysis of intrinsic characteristics of the TCR repertoires for the bulk naïve CD8^+^, bulk naïve CD4^+^, naïve RTE-enriched CD31^+^CD4^+^, and naïve non-RTE CD4^+^ T cells derived from the peripheral blood of young versus elderly healthy donors, demonstrating that

1) Characteristics of TCR beta CDR3 repertoires change in both CD4 and CD8, both RTE-enriched and mature naïve CD4 T cell subsets with age.
2) Within the same age group, no significant difference is observed in characteristics of TCR repertoire between RTE-enriched and mature naïve CD4 T cell subsets.
3) TRBV and TRBJ gene segment usage also changes prominently and similarly both within RTE-enriched and mature naïve CD4 T cell subsets of different individuals.
4) Relative “publicity” (i.e., sharing between individuals) of TCR repertoires grows both within RTE-enriched and mature naïve CD4 T cell subsets with age.

The observed changes suggest functional differences of young versus middle-age/old naïve T cell TCR repertoires with respect of potential range and characteristics of recognized antigens.

## Materials and Methods

### Donors and Cell Sorting

The study was approved by the local ethics committee and conducted in accordance with the Declaration of Helsinki. All donors were informed of the final use of their blood and signed an informed consent document. The cohort included 18 healthy individuals aged 25–88 years. Individuals with previously diagnosed cancer or autoimmune disease were excluded. Peripheral blood (10–20 ml) was collected into a number of EDTA-treated Vacutainer tubes (BD Biosciences, Franklin Lakes, NJ, USA), PBMCs extracted using Ficoll-Paque (Paneco, Kirov, Russia) density gradient centrifugation with SepMate™ tubes (STEMCELL Technologies, Vancouver, BC, Canada), and stained according to manufacturer’s recommendations. Following antibodies were used: CD3-eFluor450 (eBioscience, clone UCHT1), CD45RA-FITC (eBioscience, clone JS-83), CD27-PC5 (Beckman Coulter, clone O323), CD4-PE (Beckman Coulter, clone 13B8.2), CD25-eFluor450 (eBiosciences, clone BC96), and CD31-PC7 (eBiosciences, clone WM59). T cells of interest were sorted using FACS Aria III (BD Biosciences, Franklin Lakes, NJ, USA), directly in 350 µl of RLT buffer (Qiagen) per 100,000 sorted cells. Total RNA was further isolated using RNeasy Micro kit (Qiagen) and completely used for TCR library preparation. 5′-RACE TCR beta cDNA libraries were prepared according to the previously described protocol (43, 44). See also: https://github.com/repseqio/protocols/blob/master/Human%20TCR%20alpha%20and%20beta%20RNA-based%20RACE%20protocol.md.

Libraries were sequenced with Illumina HiSeq 2000/2500, paired-end 150 + 150 nucleotides.

### TCR Beta Repertoires Profiling and Data Analysis

T-cell receptor beta CDR3 repertoires were extracted using MiXCR software (45), version v2.1.5. Decontamination from memory T cell TCR beta clonotypes and comparative post-analysis were performed using VDJtools software v1.1.7 (46).

Resulting decontaminated TCR beta CDR3 repertoires are available from Figshare:

https://figshare.com/articles/Naive_CD4_CD8_subsets/6548921;
https://figshare.com/articles/naive_RTE_and_non-RTE_CD4_T_cells_subsets/6549059.

The obtained repertoires were further filtered to eliminate out-of-frame and stop codon-containing TCR beta CDR3 variants. Averaged physicochemical properties of amino acid residues in the middle portion (5 amino acid residues) of TCR beta CDR3 were calculated using VDJtools, the following metrics were used: strength (47, 48), hydropathy, polarity, and volume (values available from: http://www.imgt.org/IMGTeducation/Aide-memoire/_UK/aminoacids/IMGTclasses.html). During calculation, property values were weighted by the frequency of corresponding clonotypes, so the results favor more frequent clonotypes and do not depend on the sequencing/sampling depth (49). See Table 1 for the values used for each amino acid property. See Tables 2 and 3 for the counts of sorted T cells, the number of CDR3 containing sequencing reads, and the number of unique TCR beta CDR3 clonotypes in each sample.

**Table 1:** Values used for CDR3 amino acid properties calculation by VDJtools.

Amino acid	Hydropathy	Polarity	Volume	Strength
A	1.8	0	67	0
C	2.5	0	86	1
D	−3.5	1	91	0
E	−3.5	1	109	0
F	2.8	0	135	1
G	−0.4	0	48	0
H	−3.2	1	118	0
I	4.5	0	124	1
K	−3.9	1	135	0
L	3.8	0	124	1
M	1.9	0	124	1
N	−3.5	1	96	0
P	−1.6	0	90	0
Q	−3.5	1	114	0
R	−4.5	1	148	0
S	−0.8	1	73	0
T	−0.7	1	93	0
V	4.2	0	105	1
W	−0.9	0	163	1
Y	−1.3	1	141	1

**Table 2:** CD4 and CD8 naïve and memory cell sorting.

Donor	Age	Group	Subset	Replica	Number of sorted cells	Number of CDR3 reads	Number of CDR3 clonotypes
Donor 1	25	Young	CD4	1	500,000	528,824	38,614
				2	500,000	475,392	240,80
			
			CD8	1	500,000	564,227	26,988
				2	500,000	648,821	19,338
			
Donor 2	26		CD4	1	135,000	1,069,588	45,098
			
			CD8	1	251,000	633,854	28,967
			
Donor 3	35		CD4	1	200,500	380,253	43,526
				2	231,000	223,661	23,038
			CD8	1	350,500	533,091	47,626
			
Donor 4	32		CD4	1	560,000	17,494,455	165,184
				2	488,000	12,525,047	113,333
				3	395,000	6,077,772	54,546
				4	100,000	4,353,149	24,582
			
			CD8	1	510,000	9,219,066	101,096
				2	316,000	10,594,890	61,022

Donor 5	51	Old	CD4	1	495,000	13,765,788	183,873
				2	425,000	5,146,822	64,134
				3	437,000	3,832,144	84,458
				4	255,000	4,773,131	90,459
			
			CD8	1	509,000	7,369,930	125,339
				2	200,000	2,689,594	64,907
				3	343,000	517,265	20,870
				4	120,000	1,945,472	32,259
			
Donor 6	88		CD4	1	500,000	779,559	65,515
			
			CD8	1	60,000	142,639	9,991
			
Donor 7	51		CD4	1	100,000	28,129	5,033
				2	100,000	37,556	5,455
				3	100,000	41,380	6,762
			
			CD8	1	105,000	51,463	7,434
			
Donor 8	82		CD4	1	100,000	46,472	8,318
				2	90,000	38,109	6,694
			
			CD8	1	12,000	6,013	846
			
Donor 9	55		CD4	1	100,000	82,691	7,768
				2	100,000	27,215	4,740
			
			CD8	1	100,000	51,742	5,290
				2	100,000	84,532	7,666
Donor 10	77		CD4	1	254,000	677,587	24,652
			
			CD4	2	227,000	963,394	20,087
			
			CD8	1	50,000	942,430	15,033
			
			CD8	2	105,000	553,867	19,180
			
Donor 11	53		CD4	1	153,000	401,550	11,668
			
	53		CD8	1	101,000	242,248	6,857

*Donors, replicas, sorted cell counts, and number of extracted T-cell receptor beta CDR3 clonotypes are shown*.

**Table 3:** Recent thymic emigrants (RTEs)-enriched and mature naïve CD4 T cell sorting.

Donor	Age	Group	Subset	Replica	Number of sorted cells	Number of CDR3 reads	Number of CDR3 clonotypes
Donor 12	29	Young	RTE	1	50,000	251,199	27,208
			non-RTE	1	50,000	939,999	33,389
			
Donor 13	28		RTE	1	100,000	282,998	27,895
			non-RTE	1	100,000	620,320	34,092
			
Donor 14	31		RTE	1	50,000	144,571	30,139
			non-RTE	1	50,000	622,585	29,253
			
Donor 15	30		RTE	1	69,000	309,070	33,233
			non-RTE	1	100,000	2,844,397	63,900

Donor 7	51	Old	RTE	1	100,000	14,572	5,306
			non-RTE	1	105,000	17,926	4,030
			
Donor 8	82		RTE	1	38,000	16,033	2,835
			non-RTE	1	100,000	29,970	6,244
			
Donor 9	55		RTE	1	89,000	19,622	3,664
			non-RTE	1	189,000	20,891	5,290

*Donors, replicas, sorted cell counts, and number of extracted T-cell receptor beta CDR3 clonotypes are shown*.

### Statistical Analysis

For comparison of repertoire properties, one-sided *t*-test with unequal variances (Welch’s test) was used. Normality of the distribution of sample means was confirmed by performing Shapiro–Wilk tests, and the decision to reject the null hypothesis was made after adjusting for multiple hypothesis testing as in Benjamini–Hochberg. False discovery rate in normality testing was controlled at a level of 0.05 by setting *p*-adjusted upper bound at 0.05. *Z*-score normalization was performed by subtracting the mean value for each TRBV gene segment values and dividing by the SD. Only highly represented TRBV gene segments TRBV9, TRBV7−9, TRBV7−2, TRBV6−5, TRBV29−1, TRBV20−1, and TRBV12−3/12-4, each associated with at least 2% of CDR3 clonotypes in each sample, were taken into analysis.

## Results

### TCR Repertoires of Both CD4 and CD8 Naïve T Cells Change Properties With Aging

**Figure 1:**
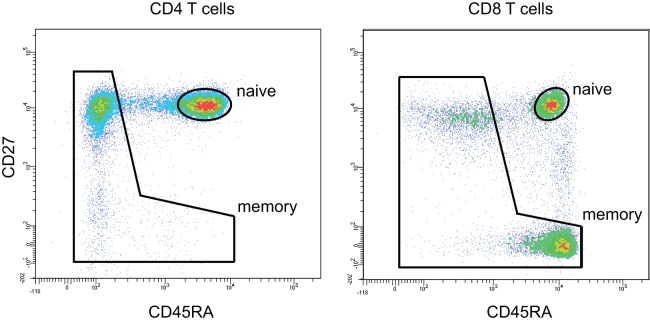
CD4 and CD8 naïve T cell gating strategy. Naïve CD4 T cells were gated as CD3^+^CD4^+^CD27^high^CD45RA^high^. Naïve CD8 T cells were gated as CD3^+^CD4^−^CD27^high^CD45RA^high^. 50,000 events were shown.

To analyze how the properties of naïve TCR repertoire change with age, we first sorted CD3^+^CD4^+^CD27^high^CD45RA^high^ and CD3^+^CD4^−^CD27^high^CD45RA^high^ T cell subsets gated as shown on Figure 1 from peripheral blood samples of 4 young (25–35 years old) and 7 middle-age/old (51–88 years old) healthy donors (Table 2). TCR beta profiling was performed as described in Ref. (43), extraction of CDR3 repertoires was performed using MiXCR (45). To exclude possible contaminations from memory T cell pool that could occur during cell sorting, we also performed TCR beta repertoires profiling for memory T cells sorted from the same donors (Figure 1). Naïve TCR beta repertoires were further decontaminated from the clonotypes present in memory subsets using VDJtools “Decontaminate” module with default parameters (20:1 parent-to-child clonotype frequency ratio for contamination filtering). This procedure eliminated from 0.005 to 0.5% of reads and from 0.01 to 0.7% of clonotypes, these numbers did not depend on the donor age group. Despite the low proportion of eliminated reads and clonotypes, such procedure is desirable for accuracy of the whole analysis and general control for cell contamination during sorting.

We complemented our data with multiplex PCR RNA-based TCR profiling data from Ref. (34) for 4 young (20–35 years) and 5 old (70–85 years) healthy donors naïve T cells gated as CD4^+^CCR7^+^CD45RA^high^CD28^+^ and CD8^+^CCR7^+^CD45RA^high^CD28^+^. Repertoire extraction was performed using the same MiXCR version starting from raw data (dbGaP, www.ncbi.nlm.nih.gov/gap, accession no. phs000787.v1.p1). Similarly, we used memory subsets from the same donors in order to decontaminate naïve T cell repertoires from possible contaminations during cell sorting using VDJtools.

**Figure 2:**
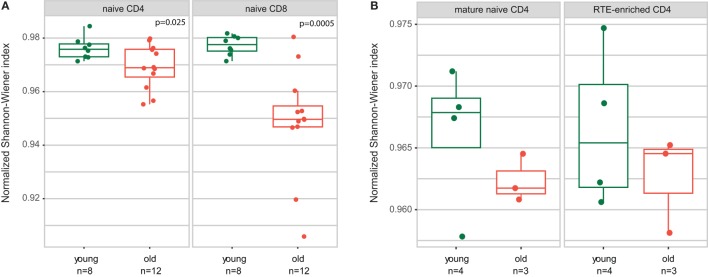
Both CD4 and CD8 naïve T cell clonality increases with human age. Normalized Shannon–Wiener diversity index for T-cell receptor beta CDR3 repertoires is shown. **(A)** CD4 and CD8 naïve T cells. Joint data from Qi and coauthors and current work. Welch Two Sample *t*-test. **(B)** Recent thymic emigrant (RTE)-enriched and non-RTE naïve CD4 T cells.

Analysis of the normalized Shannon–Wiener diversity index for the joint data confirmed the conclusion by Qi and coauthors that both CD4 and CD8 naïve T cells accumulate clonal expansion with aging (Figure 2A). The accuracy of the results for young individuals generally confirmed the validity of combining the data from both experiments, in spite of the fact that different gating was used for the naïve T cell sorting in the two studies.

Multiplex PCR employed in Qi et al. (34) may cause quantitative biases due to the differing efficiency of primers used to amplify different TRBV segments (50, 51). However, such source of bias does not influence the relative frequency of clonotypes within a particular TRBV segment. Therefore, in order to properly join our 5′RACE and multiplex PCR data from Qi et al., we performed further analysis separately for each of the TRBV gene segments that were abundantly represented in the data.

Notably, this approach has two additional benefits. First, different TRBV genes carry distinct CDR1 and CDR2 regions that participate in TCR–pMHC interaction, and, therefore, could differently influence the averaged properties of CDR3 that we analyze below. Separate analysis of TRBV segments allows to neutralize this bias. Second, distinct TRBV genes correspond to distinct T cell subpopulations allowing for independent evaluation of their properties, that provides better statistics for limited donor cohorts. All analyses were performed “weighted”—per CDR3-covering sequencing read, i.e., accounting for the relative frequency of each clonotype, with *Z*-score normalization used to combine information from different TRBV segments.

**Figure 3:**
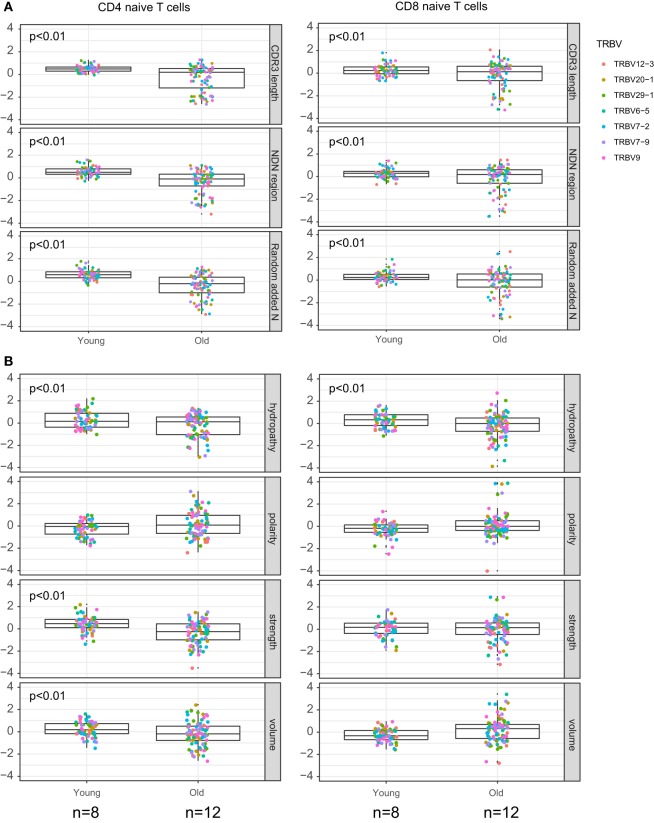
Properties of naïve T cell T-cell receptor (TCR) beta CDR3 repertoires and aging. Weighted (accounting for clonotype size) analysis of TCR beta repertoires properties for CD4 and CD8 naïve T cells derived from peripheral blood samples of young and old healthy donors. **(A)** Average CDR3 length, size of NDN insert, and count of randomly added N nucleotides. **(B)** Amino acid composition within 5 amino acid residues in the middle of CDR3. Our data and Qi et al. data, *n* = 8 young and 12 old individuals totally. CDR3 repertoires for the seven largest TRBV segments were analyzed separately, with *Z*-score normalization to account for TRBV-specific differences.

The results of comparative analysis of TRB CDR3 repertoire properties with VDJtools software are shown on Figure 3. Notably, dispersion of all parameters grows prominently with age, which already reflects the non-uniform proliferation of naïve T cells with age.

CDR3 length, size of NDN insert, and number of randomly added N nucleotides significantly decrease with age both for CD4 and CD8 naïve T cells (Figure 3A). Average characteristics of amino acid residues in the middle of CDR3 also change prominently for CD4 naïve T cells (Figure 3B).

### Both RTE and Mature Naïve CD4 T Cells Change Their Properties With Aging

To some extent, both CD45RA^+^CD31^−^ mature naïve CD4^+^ T cells and RTE-enriched CD45RA^+^CD31^+^ subsets may support their counts by peripheral division: “*CD45RA*+*CD31*+*CD4*+ *subset also undergoes some in vivo proliferation without immediate loss of CD31, resulting in an accumulation of CD45RA*+*CD31*+ *proliferative offspring* ” (30). Nevertheless, counts of CD45RA^+^CD31^+^ naïve CD4^+^ T cell notably decrease with time (5, 30). The CD31^−^ subset is believed to proliferate and support their counts more efficiently than CD31+, although the extent of telomere shortening with aging is prominent and comparable for both subsets (30).

Therefore, one could suggest that characteristics of mature naïve CD4^+^CD31^−^ T cells could change more prominently than those of RTE-enriched CD4^+^CD31^+^ T cell pool. The properties of total naïve CD4^+^ T cells could change with aging because of the intrinsic differences between the properties of RTE-enriched and mature naïve CD4 T cell TCR repertoires, and decrease of CD31^+^ cell proportion of all naïve CD4 T cells (5).

**Figure 4:**
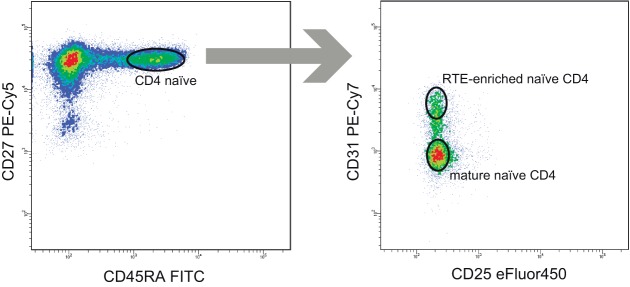
Recent thymic emigrant (RTE)-enriched and non-RTE naïve CD4 T cell gating strategy. 50,000 events were shown on the left panel.

To verify the latter hypothesis, we compared TCR beta repertoire characteristics for the sorted CD4^+^CD45RA^high^CD27^high^CD31^+^ and CD4^+^CD45RA^high^CD27^high^CD31^−^ T cells of 4 young (29–31 years) and 3 elder (aged 51, 55, and 82 years) healthy donors (Table 3). Importantly, to exclude the potential influence of naïve Tregs which characteristics essentially differ from conventional CD4 T cells, here we gated out the CD25^+^ cells from all subsets (Figure 4). It should be noted that this strict gating could also cutoff the CD25^dull^ subset of naïve CD4 T cells that was recently reported to accumulate with aging (52), however, these cells were nearly absent (represented less than 2% of naïve CD4 T cells) in our donors.

**Figure 5:**
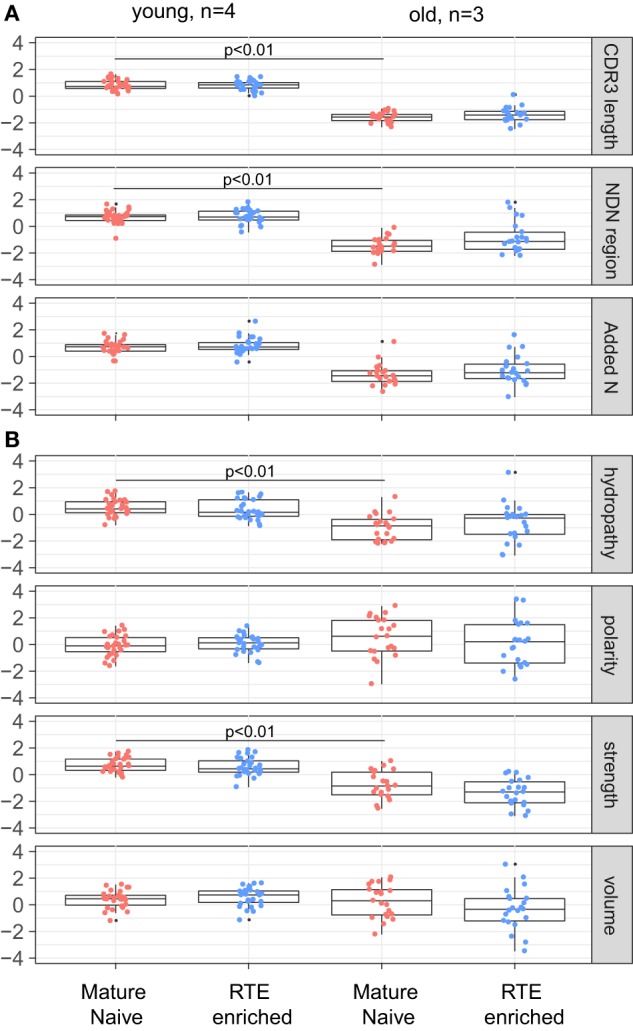
T-cell receptor beta CDR3 repertoire properties for mature naïve and recent thymic emigrant (RTE)-enriched CD4 T cells. **(A)** Average CDR3 length, size of NDN insert, and count of randomly added N nucleotides. **(B)** Amino acid composition within 5 amino acid residues in the middle of CDR3. CDR3 repertoires for the seven largest TRBV segments were analyzed separately, with *Z*-score normalization to account for TRBV-specific differences.

Analysis of obtained TCR beta CDR3 repertoires revealed that characteristics of CD4^+^CD45RA^high^CD27^high^CD25^−^CD31^+^ and CD4^+^CD45RA^high^CD27^high^CD25^−^CD31^−^CD4 T cell TCR repertoires are nearly identical within the same age group, but both prominently differ between the younger and elder donors (Figures 5A,B). It should be noted that, since the average CDR3 length decreases with age, larger portions of TRBV and TRBJ segments could be covered by our analysis of the middle 5 amino acid residues of CDR3, which could in turn influence the result amino acid property averages. However, this influence was not prominent since different TRBV segments behaved similarly in our analysis.

**Figure 6:**
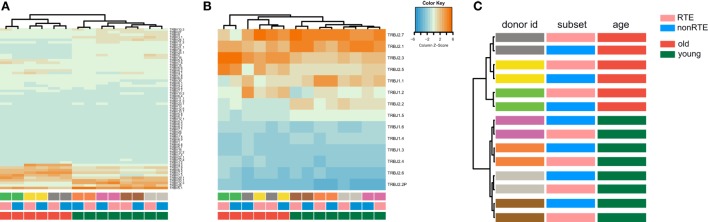
TRBV and TRBJ gene segment usage in recent thymic emigrant-enriched and mature naïve CD4 T cells of young and old individuals. **(A)** Heatmap and hierarchical clustering dendrogram for weighted TRBV usage. **(B)** Weighted TRBJ usage. **(C)** Hierarchical clustering of TRBV–TRBJ combination frequency (Jensen–Shannon divergence is used as metric). Note that repertoires of young and old individuals cluster separately.

Furthermore, young and old naïve CD4 T cell repertoires were characterized by distinct frequencies of TRBV (Figure 6A), TRBJ (Figure 6B), and paired TRBV–TRBJ (Figure 6C) gene segment usage, without any notable differences observed between the RTE-enriched CD31^+^ and mature naïve CD4 T cell subsets.

Similarly to naïve CD4 and CD8 subsets, RTE-enriched and mature naïve CD4 subsets showed a tendency toward increased clonality in the elder age (Figure 2B).

We concluded that observed changes in the characteristics of naïve CD4 T cell TCR beta CDR3 repertoire with aging affect both RTE-enriched and mature subsets, and do not result from the changes in CD31^+^/CD31^−^ subsets ratio.

### Publicity of Naïve CD4 T Cell Repertoire Grows With Aging

**Figure 7:**
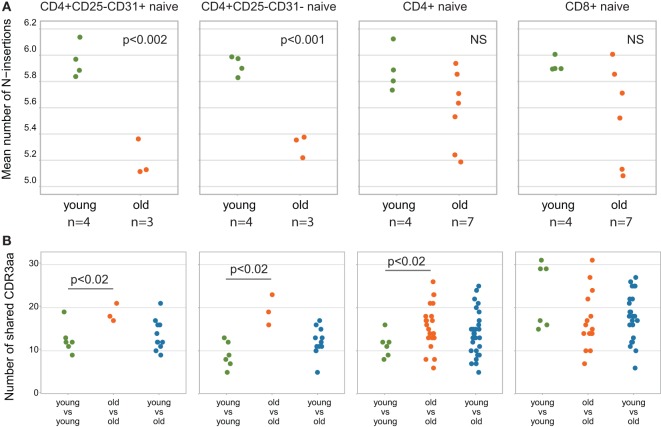
Relative publicity of naïve T cell T-cell receptor (TCR) beta repertoires. **(A)** Mean number of N-insertions among top-3,000 clonotypes from each sample. Unweighted (per clonotype), only for the clonotypes where TRBD segment borders were identified. **(B)** Repertoire overlaps calculated as the number of TCR beta amino acid CDR3 clonotypes shared between the top-3,000 clonotype repertoires for each pair of individuals. Each dot represents the number of clonotypes shared between a pair of samples. Welch Two Sample *t*-test *p*-values are shown.

Shorter CDR3 length and lower number of randomly added N nucleotides are commonly associated with higher publicity of TCR repertoires (53, 54). To analyze how the relative publicity of naïve CD4 TCR beta repertoires changes with aging, we extracted top-3,000 clonotypes from each dataset, with random sampling of clonotypes having the identical low frequency—a normalization step which is highly desirable to minimize biases in comparison of immune repertoires overlaps. As it could be expected based on CDR3 characteristics (Figures 5A and 7A), analysis of relative overlaps between TCR beta CDR3 repertoires revealed that relative publicity of total CD4 naïve [our data only, excluding the data from Ref. (34)], RTE-enriched CD31^+^ and mature naïve CD31^−^ CD4 T cell subsets grows with aging (Figure 7B). A moderate overlap was observed between the young and middle-age/old CD4 naïve, RTE-enriched CD31^+^ and mature naïve CD31^−^ CD4 T cell subsets. No clear age-related changes in relative publicity were observed for CD8 naïve T cells (our data only).

We used CDR3 sequence similarity graph to analyze whether naïve TCR repertoires form separate networks in young versus old donors. To build the graph, we selected 3,000 most abundant clonotypes from each donor and pooled them together to form the set of nodes. We connected two clonotypes with an edge if they had the same VJ-combination and CDR3 differed by a single amino acid substitution. Next, we counted the number of edges connecting clonotypes from donors of different age groups (young versus old) and obtained empirical distributions for these counts by running 1,000 random permutations of age group labels.

We found, that in CD8 naive repertoires, the number of edges between clonotypes from young and old donors is larger in data than in simulation in 424 donor age group permutations out of 1,000, so there is no evidence for separate CDR3 networks for young and old donors for this subset. In CD4 naive repertoires, however, there was a weak tendency: only in 95 simulations out of 1,000 (empirical *P*-value of 0.095) we found a lower number of edges between donors of different age, than the one observed in real data. This suggests that repertoires of naive CD4 T cells include distinct communities of homologous TCR variants in young and old individuals. However, this effect was only marginally significant and requires further investigation.

## Discussion

With aging, decreasing thymic output can not efficiently sustain naïve T cell counts, so the homeostatic proliferation becomes the main mechanism to replenish this cell pool in humans. Such proliferation is inevitably associated with certain biases that shape the landscape of naïve T cell TCR repertoire and thus affect the spectrum of the antigens they could recognize.

We have utilized immune repertoire sequencing to study the repertoires of naïve T cells in young and aged donors and revealed notable changes in human TCR repertoires of both CD4 and CD8 peripheral blood naïve T cells with aging:

(1) We confirm the observation of Ref. (34) that relative clonality reflecting the extent of clonal expansion increases both within CD4 and CD8 naïve T cell subsets with age (Figure 2).
(2) We demonstrate that average CDR3 length, NDN insert length, and number of randomly added N nucleotides significantly decrease with aging in all subsets of naïve T cells, including CD4, CD8, CD4 RTE-enriched CD25^−^, and CD4 mature naïve CD25^−^ subsets (Figures 3A, 5A and 7A). Interestingly, due to spatial restrictions in TCR–pMHC interaction, the length of CDR3 is inversely related to the length of recognized peptide antigen, which affects the spectrum of recognized pMHCs (Shugay et al., manuscript under consideration). The decrease of CDR3 length with aging could, therefore, reflect the averaged properties of pMHCs that are preferentially recognized by naïve T cells in the periphery, and cause better tonic signaling, leading to earlier exhaustion of proliferation capacity of the cells carrying corresponding TCR variants.
(3) As could be expected based on previous works (53, 54), the abovementioned changes favored higher publicity in CD4 naïve T cells (Figure 7B). At the same time, we have not observed clear differences in TCR beta CDR3 repertoire publicity for CD8 compartment. These observations differ from the data from Qi et al. (34) suggesting the decrease of CD8 naïve T cell publicity with aging. Further studies on larger cohorts with thoroughly controlled purity of cell sorting, and proper normalization of the datasets for comparing publicity of repertoires (49) should clarify this point.
(4) Averaged amino acid characteristics in the middle of CDR3 change prominently in CD4, CD8, CD4 RTE-enriched, and CD4 mature naïve subsets (Figures 3B and 5B). In particular, significant decrease is observed for the “strength” metrics, which represents the count of strongly interacting amino acid residues (47, 48). The “strongly interacting” include F, L, I, M, and V that may form hydrophobic contacts, as well as aromatic residues W and Y that are capable of different types of interactions including offset stacked or edge-to-face interactions, thiol–aromatic interactions, and others (55), and may consist of electrostatic, van der Waals, and hydrophobic forces. Correspondingly, similar changes are observed for the “hydropathy” metrics which counts the number of hydrophobic residues in the middle of CDR3.

**Figure 8:**
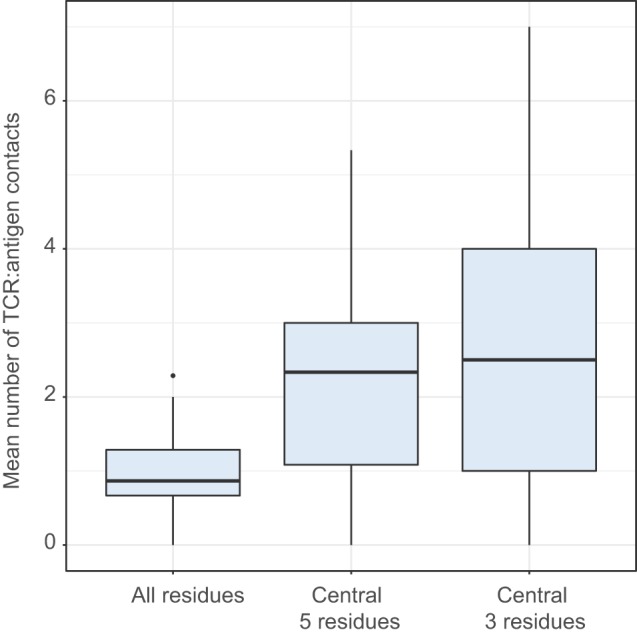
Number of CDR3:antigenic peptide contacts in structural data. Comparing the mean number of contacts for entire CDR3 (All positions) and its central region (central 5 residues and central 3 residues). ANOVA followed by a *post hoc* Tukey test shows significantly higher number of contacts for the central region: *P* < 10^−8^ when comparing 5 and 3 central residues to all residues, but no difference between 5 and 3 central residues (*P* = 0.42). The analysis was performed for T-cell receptor (TCR) beta chain using 110 human TCR:pMHC complexes from Protein Data Bank.

The “strength” metric efficiently differentiates functional T cell subpopulations, such as Treg and non-Treg CD4 subsets [see Ref. (49, 56) and our data to be published elsewhere]. This metric can be interpreted as an averaged estimation of TCR repertoire affinity to peptide–MHC complexes and in particular to the antigenic peptide, since the middle portion of CDR3 is often in contact with the presented antigen (Figure 8).

The decrease of relative abundance of strongly interacting amino acid residues within TCR beta CDR3 repertoire of naïve T cells with aging may, therefore, reflect more rapid depletion of naïve T cell clones with higher affinity to self pMHC. This could result from more efficient tonic signaling and generally faster proliferation, exhaustion of proliferation capacity, and extinction of such naïve T cells (38).

Notably, similar changes were observed within RTE-enriched CD31^+^ and mature naïve CD31^−^ CD4 naïve T cells (Figures 5–7). Decrease of the “strength” metric was even more prominent for the RTE-enriched subset (Figure 5B), suggesting that the CD31^+^ naïve CD4 T cell clones bearing TCR variants with high affinity to self pMHC are prominently switching to the CD31^−^ phenotype due to more efficient TCR signaling.

Complementary explanation for the changes observed in the naïve T cell TCR repertoire characteristics with aging is that the high affinity variants are washed away from the naïve T cell pool in the course of ongoing immune responses. Both CD4^+^ and CD8^+^ T cells with strong reaction to self and high tonic signaling dominate in responses to foreign antigens (37, 57, 58). Positive selection in thymus thus favors production of more efficiently responding T cells that should be also more rapidly depleted from the naïve T cell pool. If this is the case, the age-related changes are associated with generation of prominent functional holes in the landscape of naïve T cell receptor repertoire.

An additional factor that could contribute to the observed changes in naïve T cell TCR repertoires is the easier conversion of clones with high affinity to self pMHC to the “memory-like” phenotype, as shown in mice models (59, 60), although such observations have not yet found clear confirmation in humans (3).

Altogether, the observed changes could be interpreted as elimination of generally more “sticky”—having higher affinity to self and non-self peptide–MHC complexes—TCR variants from the naïve T cell pool with aging.

However, there is also an alternative explanation which deserves consideration. Shorter CDR3s, lower numbers of randomly added N nucleotides, and higher publicity are characteristic features of the early wave(s) of naïve T cells generated during fetal period (23, 40, 61–63). Such early wave(s) originate from distinct population(s) of hematopoietic stem cells that may have distinct long-term program including higher proliferation potential (39, 42).

Considering the drop in thymic activity that happens in the middle age (4, 26), one could hypothesize that the counts of conventional naïve T cell decrease after exhaustion of their limited proliferation capacity, while the early-wave naïve T cells of fetal origin with prolonged proliferation capacity persist. Such organization of T cell adaptive immunity in the elderly could be beneficial from the point of more predictable innate-like behavior of the T cells carrying a relatively restricted, more germline-encoded TCR repertoire. To some extent, our network analysis of naïve CD4 T cell TCR repertoires supports this concept.

Summing up, our study sheds light on the intrinsic changes in the naïve T cell TCR repertoire structure with aging, and calls for further functional studies that could clarify the underlying mechanisms.

## Ethics Statement

The study was approved by the local ethics committee and conducted in accordance with the Declaration of Helsinki. All donors were informed of the final use of their blood and signed an informed consent document.

## Author Contributions

EE and DS performed cell sorting. EE, SK, VZ, TN, MS, and MP analyzed the data. EE, MS, and DC prepared the figures. EE, MI, AA, FC, IM, ER, and AF worked on library preparation and sequencing. DC and OB designed the entire study and wrote the manuscript. MS, ER, and AF edited the manuscript. All authors reviewed and approved the final manuscript.

## Conflict of Interest Statement

The authors declare that the research was conducted in the absence of any commercial or financial relationships that could be construed as a potential conflict of interest.
